# Retention of the posterior cruciate ligament does not affect femoral rotational alignment in TKA using a gap-balance technique

**DOI:** 10.1007/s00167-014-3218-8

**Published:** 2014-08-14

**Authors:** Yoshinori Ishii, Hideo Noguchi, Junko Sato, Koji Todoroki, Shin-ichi Toyabe

**Affiliations:** 1 Ishii Orthopaedic and Rehabilitation Clinic, 1089 Shimo-Oshi, Gyoda, Saitama 361-0037 Japan; 2 Department of Medical Informatics, Niigata University Medical and Dental Hospital, 1-754 Asahimachi, Niigata, Niigata 951-8520 Japan

**Keywords:** PCL retention, Femoral rotational alignment, TKA, Gap-balance technique

## Abstract

**Purpose:**

Previous studies have evaluated the ability of the gap technique to achieve accurate rotational placement in both posterior cruciate ligament (PCL)-retaining and PCL-substituting total knee arthroplasty (TKA). The purpose of the present study was to determine (1) the accuracy of this technique in degrees and (2) whether retention of the PCL affects the rotational alignment of the femoral component relative to the transepicondylar axis during TKA. The hypothesis of this study was that retention of the PCL does not affect the femoral rotational alignment in TKA using a gap-balancing technique because both procedures are reported to have good long-term clinical outcomes.

**Methods:**

The femoral rotation angle (FRA) relative to the transepicondylar axis was examined in 206 patients who underwent primary TKA using either PCL-retaining (104 knees) or PCL-substituting (102 knees) prostheses to determine the effect of PCL retention on FRA. Quantitative three-dimensional computed tomography was used to assess the FRA in both groups. All values are expressed as median (25th percentile, 75th percentile).

**Results:**

Postoperative FRA in the PCL-retaining group was −1.1° (−2.8°, 2.2°) and in the PCL-substituting group was −0.1° (−2.5°, 2.8°). The groups were not statistically different. One outlier was found in the PCL-retaining group, and none was found in the PCL-substituting group.

**Conclusions:**

The gap technique reliably allows accurate rotational alignment of the femoral component during TKA despite the retention of the PCL.

**Level of evidence:**

Therapeutic study, Level II.

## Introduction

A technique that consistently achieves accurate rotational alignment of the femoral component of the prosthesis relative to the transepicondylar axis during total knee arthroplasty (TKA) has yet to be determined. The two primary techniques reported in the literature are as follows: (1) a measuring technique using anatomical reference points (the femoral epicondyles, posterior femoral condyles, or the anteroposterior axis) to determine the proper rotational placement of the femoral component of the implant, and (2) the gap technique, in which the femoral component is positioned parallel to the resected proximal tibia with each collateral ligament equally tensioned.

Because the posterior cruciate ligament (PCL) is tensioned when the knee is in flexion [5] and acts as a secondary rotational stabilizer [6], it is crucial for surgeons to recognize that retention of the PCL creates a rectangular space between the femoral implant and the osteotomized tibia during knee flexion. However, most previous studies [6, 11, 18, 22, 24, 25] that used the gap technique compared the accuracy of the rotational positioning of the femoral component relative to the transepicondylar axis in either PCL-retaining [6, 11, 18] or PCL-substituting [22, 24, 25] TKA. There are no reports that have compared the effects of retention of the PCL on the rotational positioning of the femoral component of the implant. In the present study, accuracy is defined as a femoral rotation angle (FRA) between 5° of internal rotation and 5° of external rotation relative to the transepicondylar axis. Thus, the aim of this study was to determine (1) the accuracy (internal and external rotation within 5° of the transepicondylar axis with minimal outliers) achievable using the gap (soft tissue balancing) technique, and (2) whether the retention of the PCL improves or hinders the correct rotational placement of the femoral component relative to the transepicondylar axis when comparing PCL-retaining and PCL-substituting mobile-bearing TKA implants. The hypothesis of this study was that retention of the PCL does not affect the femoral rotational alignment in TKA using a gap-balance technique because both procedures are reported to have good long-term clinical outcomes [2, 10].

## Materials and methods

All patients provided informed consent. The study population consisted of patients undergoing TKA for either primary osteoarthritis or rheumatoid arthritis. Patients undergoing revision arthroplasty, or those who had previously undergone tibial osteotomy, were excluded from the study. Between March 2006 and August 2013, 222 TKAs were performed in 210 patients. All 206 patients who were eligible for inclusion agreed to participate (Table 1). All knees were implanted with the LCS^®^ Total Knee System (DePuy, Warsaw, IN, USA).

**Table 1:** Patient demographics

Variable	PCL-retaining design	PCL-substituting design
Number of knees/patients	104/104	102/102
Sex (male/female)	13/91	17/85
Diagnosis (OA/RA); knees	103/1	100/2
Age (years)^a^	71 (8)	72 (8)
BMI (kg/m^2^)^a^	26 (4)	26 (4)
Preop. coronal angle (°)	180.8 (3.6)	181.4 (5.2)
Postop. coronal angle (°)	174.2 (2.8)	174.1 (3.0)

*OA* osteoarthritis, *RA* rheumatoid arthritis, *Preop.* preoperative, *Postop.* postoperative

^a^Values are expressed as mean (SD)

The median age of the patients was 73 years (range 34–90 years) at the time of surgery. One hundred and four knees (104 patients) received meniscal-bearing-type PCL-retaining prostheses, and 102 knees (102 patients) received rotating-platform-type PCL-substituting prostheses. Complete follow-up data were obtained from all patients in this series. The treatment allocation was made by a quasi-randomized approach, using even chart numbers for the PCL-retaining group and odd chart numbers for the PCL-substituting group. The two prosthesis designs had the same geometry in the axial plane. However, the PCL-retaining design had nonconstrained anteroposterior and rotational movement, while the PCL-substituting design had only nonconstrained rotational movement. The LCS^®^ femoral component had an anatomic articulating surface with a smaller radius of curvature at the posterior aspect of the femoral condyles. The LCS^®^ femoral and tibial components were fully conforming in the sagittal plane from full extension to 30° flexion and less conforming at greater flexion angles because of this smaller radius of curvature at the femoral posterior condyles.

A single surgeon (YI) performed all the surgeries using standardized techniques as described previously [8]. Ligament-balancing techniques, which included the necessary soft tissue release and removal of peripheral osteophytes, were used and confirmed with spacer blocks to ensure a balanced knee with equal flexion and extension gaps. In the flexion gap first technique, also called the balanced flexion gap technique, the proximal tibial osteotomy was performed first. The tibial osteotomy was perpendicular to the mechanical axis of the tibia. With the knee in 90° flexion, the anteroposterior femoral cutting block was positioned relative to the anterior cortex of the femur using a femoral intramedullary alignment rod. The femoral positioner was used to make the anterior and posterior femoral resections parallel to the tibial resection. The tension of the collateral soft tissues was adjusted by adding shim plates on the tibial side of the arthroplasty if the space was too large, leaving the tissues lax. If one compartment was still too tight during flexion, additional soft tissues, including the PCL in the PCL-retaining implant group, were released to achieve equal compartmental tension. The goal in determining femoral rotation was to establish a quadrilateral space, with the resected surfaces of the posterior femoral condyles parallel to the resected tibial surfaces when the collateral ligaments were tensioned. The distal femoral cuts were done last, using an intramedullary guide to create neutral alignment and balance with the desired tension in extension. Ultimately, the knee had equivalent rectangular gaps at both 90° and 0° of flexion. The trial prosthesis was inserted and checked before placing the final prosthesis. Although no intraoperative quantitative evaluation was performed, the proper intraoperative coronal and sagittal plane laxity was confirmed manually. As reported in our previous study, achieving about 4° of coronal laxity in extension and 3° of coronal laxity in flexion, measured using an arthrometer, was associated with good clinical outcomes for both prostheses [14].

**Fig. 1:**
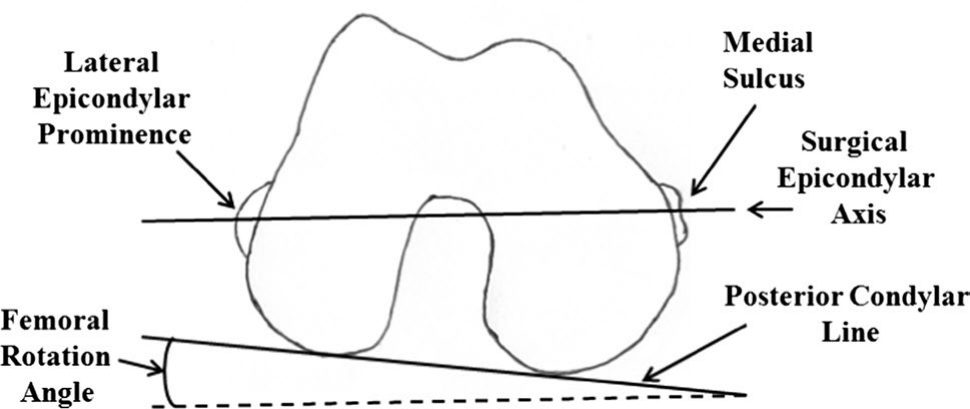
Definition of femoral rotation angle (FRA). Schematic of the axial view of the right distal femur as seen from below by the surgeon during total knee arthroplasty with the knee flexed at 90°. FRA is the angle between the posterior condylar surfaces and the surgical axis, defined using the medial sulcus on the medial epicondyle

A quantitative three-dimensional (3D) technique developed by Sato et al. [16, 17] which uses the posterior condylar line and transepicondylar axis to evaluate posterior condylar angle, was used. This assessment required acquisition of preoperative CT images of each patient’s femur and tibia. In addition, biplanar computed radiography (CR) images of the lower extremities were obtained before and 3 weeks after TKA. The biplanar CR images were transferred to a personal computer running a 3D lower extremity alignment assessment system (Knee CAS, LEXI, Inc., Tokyo, Japan). The 3D digital bone and component models were projected onto the biplanar CR images using the camera calibration technique. Matching the silhouettes of these digital models to the contours of the respective bone images and component CR images through 3D rotation and translation allowed computation of the 3D position and alignment of the components relative to the femur and tibia. After these image-matching procedures, a 3D view of the digital model complex was displayed in which the component models were implanted into the bone models. Any rotation between various points in the 3D digital model could be computed, and a cross-sectional view of the 3D digital model complex could be displayed for any plane. Additional details describing this system have been published previously [1, 13, 16, 17]. The obtained CT and CR data were used to define the posterior condylar line and surgical transepicondylar axis of the femur. The posterior condylar line was defined as the line connecting the edge of each condyle. The surgical transepicondylar axis was defined as the line connecting the lateral epicondylar prominence and the medial sulcus on the medial epicondyle (Fig. 1). To minimize inter-observer variation, a single experienced technician (KT) performed all the tests. The maximum spatial error of this procedure was 0.8° when determining rotation using the current system [16]. The calculation of rotation was highly reproducible, with a maximum intra-observer error of 1.2°, including all analytical processes.

**Fig. 2:**
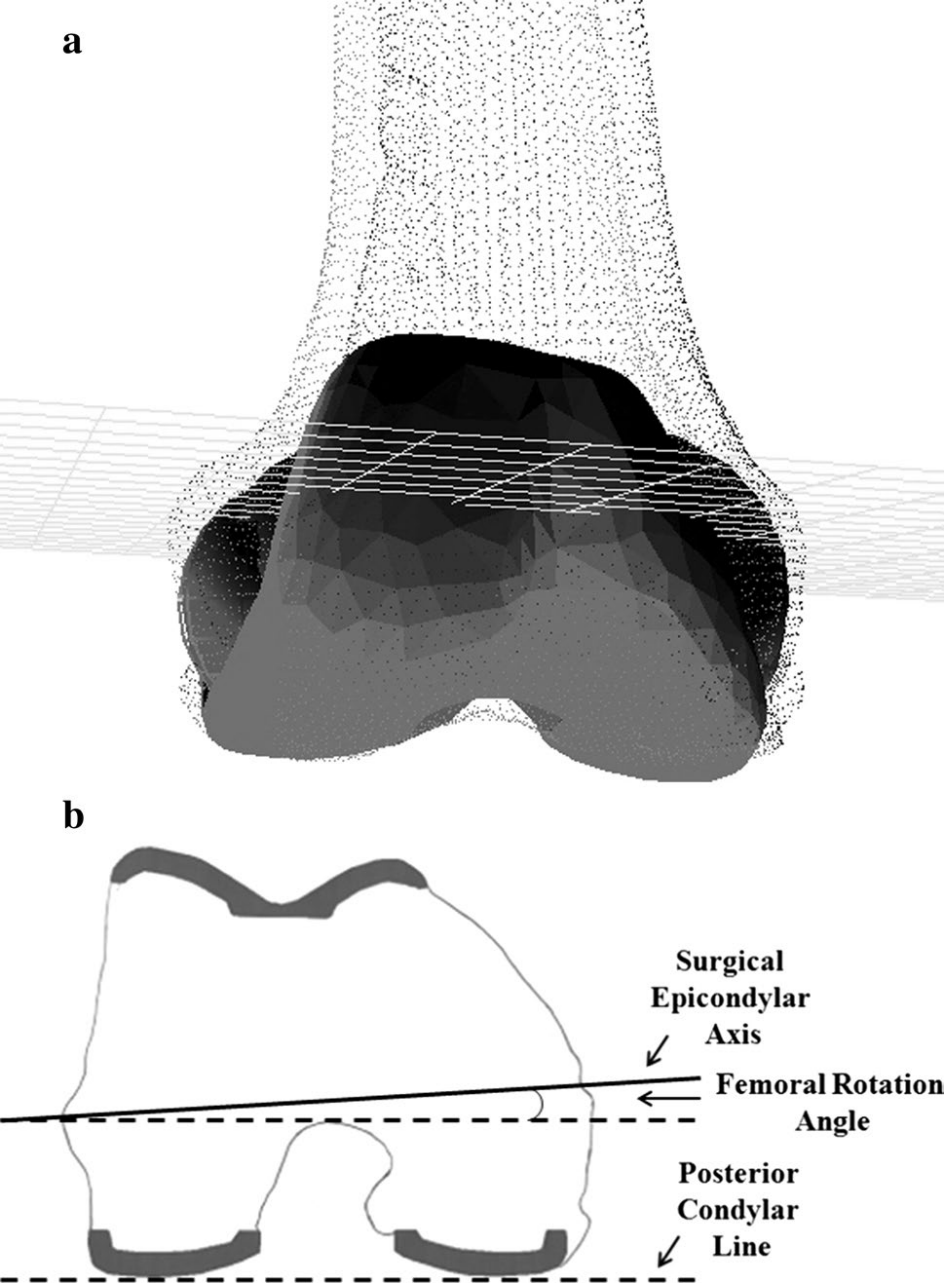
**a**, **b** Cross-sectional views in the axial plane of the femoral component of a prosthesis used in TKA are shown. **a** A digital model of the prosthesis complex is shown. **b** Measurement of the postoperative femoral rotation angle (FRA) that is the angle between posterior condylar line (*dotted line*) and transepicondylar axis

The postoperative surgical transepicondylar axis and posterior condylar line were measured on 3D cross-sectional views in the axial plane for the postoperative femur and the femoral component. The FRA, which is the angle between the posterior condylar line (the posterior condylar surfaces) and the transepicondylar axis, was compared between PCL-retaining and PCL-substituting implants (Fig. 2).

**Fig. 3:**
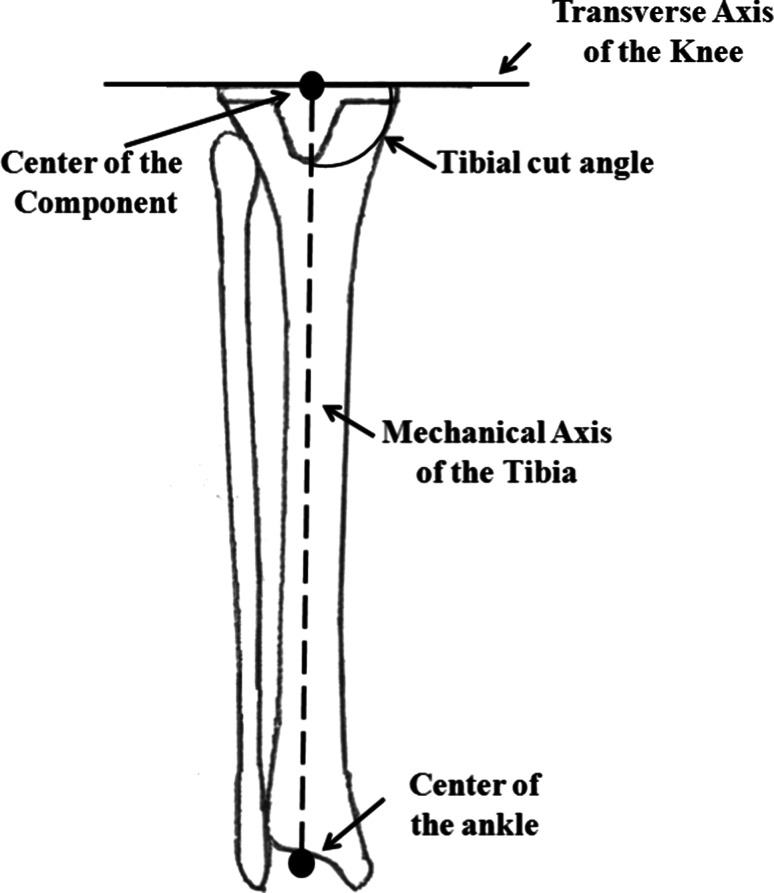
Definition of tibial cut angle

In addition, the tibial cut angle (TCA) was defined as the angle between the mechanical axis of the tibia and the transverse axis of the knee joint after TKA using anteroposterior CR images of the lower extremities (>90°: valgus; 90°: neutral; <90°: varus) (Fig. 3). Finally, all values were expressed as negative (−) internal rotation relative to the transepicondylar axis and positive (+) external rotation relative to the transepicondylar axis. Approval for this study was obtained from the Research Board of Healthcare Corporation Ashinokai, Gyoda, Saitama, Japan.

### Statistical analysis

Wilcoxon’s rank-sum tests were used to compare the TCA, the pre- and postoperative FRA, and the differences between the pre- and postoperative FRA between the two implant designs. Based on a power analysis, the 104 samples in the PCL-retaining and 102 samples in the PCL-substituting designs were determined to be sufficient to detect a correlation coefficient of 0.5 with 91.2 % power when analyzed by a nonparametric approach (Wilcoxon’s rank-sum test) with a two-sided *α* error of 5 %. All values are expressed as medians (25th percentile, 75th percentile). Statistical analyses were performed using IBM SPSS Statistics ver. 20 (IBM Japan, Ltd., Tokyo, Japan). For all tests, *p* values less than 0.05 were considered statistically significant.

## Results

The postoperative FRA in the PCL-retaining implant group was −1.1° (−2.8°, 2.2°) and in the PCL-substituting implant group was −0.1° (−2.5°, 2.8°). These values were not significantly different (*P* = 0.386) between groups. The FRA data ranges are summarized in Table 2. The range of FRA was 10.8° (4.9° internal rotation to 5.9° external rotation) in the PCL-retaining implant group and 9.9° (4.9° internal rotation to 5.0° external rotation) in the PCL-substituting group. One unacceptable surgical outlier, defined as outcomes with more than 5° rotation from the reference transepicondylar axis [25], was found in the PCL-retaining implant group, and no outliers were found in the PCL-substituting group.

**Table 2:** Preoperative femoral rotation angle (FRA), postoperative FRA, and the differences of pre- and post-FRA in each design

Variables range	Preoperative FRA	Postoperative FRA	Differences in FRA
Retaining^a^ (*n* = 104)	−0.5° (−1.3°, 0.5°) [− 3.8°, 3.5°]	−1.1° (−2.8°, 2.2°) [− 4.9°, 5.9°]	−0.2° (−2.4°, 2.5°) [− 6.0°, 7.1°]
Substituting^a^ (*n* = 102)	−0.4° (−1.0°, 0.3°) [− 3.6°, 3.2°]	−0.1° (−2.5°, 2.8°) [− 4.9°, 5.0°]	0.3° (−2.2°, 3.0°) [− 5.7°, 7.0°]
*p* value	n.s.	n.s.	n.s.

All values are expressed using minus (−) internal rotation relative to transepicondylar axis and plus (+) external rotation relative to transepicondylar axis

*FRA* femoral rotation angle

^a^Values are expressed as median (25th percentile, 75th percentile)

In addition, the differences between the pre- and postoperative FRA were −0.2° (−2.4°, 2.5°) in the PCL-retaining implant group and 0.3° (−2.2°, 3.0°) in the PCL-substituting implant group, which were not significantly different (n.s.).

The TCA was 89.2° (87.7°, 90.8°) (range 85.2–94.6°) in the PCL-retaining implant group and 89.8° (88.0°, 91.3°) (range 85.1–94.4°) in the PCL-substituting implant group. These values were not significantly different (n.s.). The preoperative FCA values were also not significantly different between the groups (n.s.) (Table 2).

## Discussion

These data indicate that: (1) both implant designs showed comparable or greater accuracy of rotational placement compared with previous studies using the gap technique [6, 11, 18, 22–25], and (2) there were no statistical differences in the accuracy of femoral component rotational placement between mobile-bearing PCL-retaining and PCL-substituting prostheses. For the current implant designs, the gap technique allowed reliable and accurate rotational placement of the femoral component during TKA when the transepicondylar axis is defined as the optimal reference axis.

One limitation of this study is that the results cannot be generalized to all knee arthroplasty patients because only mobile-bearing designs were evaluated. The PCL-retaining and -substituting femoral prostheses used here have the same geometry in medial and lateral condyle. Changes in the conformity of the femoral and tibial coupling with flexion should be carefully examined in these prostheses, as well as in those with a femoral component design having a single radius and an asymmetric medial and lateral condylar design. Another limitation is that the clinical significance of PCL retention with regard to the rotation of the femoral component relative to the transepicondylar axis was not evaluated in the different implant designs, only the accuracy of the FRA using the gap technique. However, no significant differences in the clinical results [9] or the coronal soft tissue balance in extension [7] and flexion [14] of knee arthroplasties using both implant designs were found in our previous studies using the same procedure performed by the same surgeon (YI).

The accuracy of the FRA using the gap technique has been reported as between 0.6° and 4.4° relative to the transepicondylar axis or the plane of the tibial osteotomy, and the ranges of internal to external rotation were between 5.4° and 26° [6, 11, 18, 22–25] (Table 3). In the present study, the median value of the FRA in PCL-retaining designs was 1.1° and in PCL-substituting designs was 0.1°, with an approximately 10° range and no significant difference between the implant designs. In addition, only one out of 104 cases in which the PCL-retaining implant was used showed over 5° of FRA error. Several factors may have contributed to these similar and good results for both designs. First, there were no preoperative anatomical differences in the FRA between the implant groups. Second, the same perpendicular cut to the tibial anatomical axis was used in both groups. Finally, the patients were treated by a single, experienced surgeon (YI) using the same instrumentation in all cases. Two previous studies [15, 20] reported inter-surgeon variability in the accuracy of the rotational setting of the component for both computer-assisted and conventional TKA. Although achieving an accurate alignment may depend on the skill of the surgeon, the gap technique results in an optimal rotational placement of the femoral component of the implant more reliably than the measuring technique [18, 22, 23, 25] because it does not rely on bony landmarks [12, 21].

**Table 3:** Previously reported values for the accuracy of femoral rotation angle

Authors	Mean	IR	ER	Range	>5° error	PCL
Heesterbeek et al. [6]^a^	4° (4.3°)	4°	13°	17°	51 % >3°	+
Kaipel et al. [11]	2.5°	3°	7°	10°		+
Schnurr e al [18]^a^	4.4° (3.7°)	11.5°	11.8°	23.8°		+
Vaidya et al. [22]^a^	2.67° (1.11°)					−
Walde et al. [23]^b^	0.6° (0.07°)	3.0°	2.4°	5.4°		^c^
Witoolkollachit et al. [24]^a^	2.39° (2.80°)	8.29°	2.22°	10.51°		−
Yau et al. [25]^a^	0.8° (3°)	14°	12°	26°	20 %	−

*IR* internal rotation, *ER* external rotation

^a^Values are expressed as mean (SD)

^b^Values are expressed as mean (SE)

^c^No description

Several advantages of using the gap-balancing technique [3] support the results of the present study. Further studies using a combination of the gap technique and the measuring technique, as suggested by Dennis et al. [4] as a secondary check and which may already be used by many surgeons, could further improve femoral component placement precision relative to the transepicondylar axis. A study of the etiology of total knee revision found that early failure mechanisms are primarily surgeon-dependent [19]. Thus, improving surgeon technique may yield even better rotational alignment of the femoral component than the results presented here.

## Conclusion

Retention of the PCL does not affect the accuracy of femoral rotation alignment with the gap technique in the current design of mobile-bearing TKA. Both PCL-retaining and -substituting designs with the gap technique resulted in comparable or better femoral rotational alignment compared with previous reports [6, 11, 18, 22–25]. Therefore, based on the accuracy of femoral rotational alignment relative to the transepicondylar axis, the gap technique appears to be a reliable procedure for both PCL-retaining and -substituting designs.
